# A Tube Model Predictive Control Method for Autonomous Lateral Vehicle Control Based on Sliding Mode Control

**DOI:** 10.3390/s23083844

**Published:** 2023-04-09

**Authors:** Yong Dai, Duo Wang

**Affiliations:** 1 School of Automation and Electrical Engineering, Shenyang Ligong University, Shenyang 110159, China; 2 Science and Technology Development Corporation, Shenyang Ligong University, Shenyang 110003, China

**Keywords:** autonomous vehicle (AV), model predictive control (MPC), sliding mode control (SMC), tube MPC, path tracking

## Abstract

This paper aims to enhance the lateral path tracking control of autonomous vehicles (AV) in the presence of external disturbances. While AV technology has made significant strides, real-world driving scenarios often pose challenges such as slippery or uneven roads, which can adversely affect the lateral path tracking control and reduce driving safety and efficiency. Conventional control algorithms struggle to address this issue due to their inability to account for unmodeled uncertainties and external disturbances. To tackle this problem, this paper proposes a novel algorithm that combines robust sliding mode control (SMC) and tube model predictive control (MPC). The proposed algorithm leverages the strengths of both MPC and SMC. Specifically, MPC is used to derive the control law for the nominal system to track the desired trajectory. The error system is then employed to minimize the difference between the actual state and the nominal state. Finally, the sliding surface and reaching law of SMC are utilized to derive an auxiliary tube SMC control law, which helps the actual system keep up with the nominal system and achieve robustness. Experimental results demonstrate that the proposed method outperforms conventional tube MPC, linear quadratic regulator (LQR) algorithms, and MPC in terms of robustness and tracking accuracy, especially in the presence of unmodeled uncertainties and external disturbances.

## 1. Introduction

The main technologies of an autonomous vehicle (AV) typically include environmental perception, behavioral decision-making, path planning, and motion control. To ensure safe, smooth, and comfortable driving of an AV, the motion control algorithm has become a top priority in modern autonomous driving technology [1]. The motion control of an AV is typically divided into two categories: longitudinal control and lateral control [2]. Longitudinal control focuses on controlling the speed and distance between vehicles, which has been thoroughly resolved in recent years. On the other hand, lateral control is responsible for steering the vehicle and ensuring it stays on a predetermined path, even in the presence of internal unmodeled uncertainty and external disturbances like slippery or rough roads [3,4], which has yet to be fully resolved. Currently, the most commonly used lateral control methods for AV include pure tracking control, proportional-integral-derivative (PID) control, model-free control, linear quadratic regulator (LQR), feed-forward control, sliding mode control (SMC), H∞ control, and model predictive control (MPC), among others [5,6].

For example, Zhao et al. [7] designed a pure tracking control method based on dynamic delay prediction to obtain sight control by using the deviation value between the travel direction and the tracking direction. Kapsalis et al. [8] combined linear parameter varying (LPV) control theory with a new pure tracking control method to realize stable driving of vehicles with variable speed. Ahn et al. [9] proposed an improved pure tracking method to enhance the tracking accuracy of low-speed unmanned vehicles in straight lines and curves based on Ackerman’s steering geometry. These pure tracking control methods have the advantages of being simple, low-speed, and flexible, but the disadvantage is that they will be limited by road curvature conditions. Moshayedi et al. [10] proposed a method to optimize the PID controller for an AV model using PSO and BAS algorithms. The effectiveness and rapidity of the method were verified on five different paths, making it valuable for researchers in the field of service robots. In a later study, they extended remote sensing applications to calibrate drone cameras accurately, ensuring precise detection of vehicle speed to enhance the operating efficiency of vehicles in congested road environments, thereby improving intelligent city services based on the Internet of Things [11]. Chu et al. [12] proposed a trajectory-tracking framework based on the PID feedback method, with a steady-state error close to zero when finally tracking the curve. The advantage of this method is that it is easy to design for engineering applications, but the PID controller has the problem of poor performance, and the tuning of its control parameters is always a challenge in PID control [13]. Jiang et al. [14] and Wang et al. [15] proposed a simple control framework using a model-free control method for ideal road driving, but it has poor robustness and is challenging to analyze the stability of the control system as a black box. Park et al. [16] designed a feedback controller based on the LQR method, which can maintain balance and track the circle in the drift state. Najjari et al. [17] presented an LQR controller by studying the torque vectoring system and steering controller, making it easy to achieve closed-loop optimal control of tracking the target. However, these LQR methods have poor robustness as the controllers are designed based on offline calculation. Jiang et al. [18] proposed a constrained arc fitting method to design the feed-forward control model of curvature, which improves the control accuracy, and Khan et al. [19] proposed a feed-forward control method to process external disturbances, modeling error, and sensory noises. Still, these methods require expensive sensors mounted on the vehicle to collect data at a high cost, which can only be used in specific situations and are unsuitable for mass production. Wu et al. [20] used SMC to calculate the total driving force of vehicle lateral control, improving the adaptability of control algorithms and tracking accuracy at high speed. Ding et al. [21] added an improved second-order SMC with power integrator technology to improve the transient performance of path-tracking errors. However, there was a chattering problem caused by SMCs in the path tracking. Yan et al. [22] improved comfort by using an H∞ control to suppress noise while maintaining the lane based on the incremental control vehicle model, and Liu et al. [23] designed an H∞ control method according to system performance parameters to have strong robustness to sideslip angle measurement, model uncertainty, and external disturbances. However, this type of H∞ controller requires a complex solution and calculation process.

MPC is considered the simplest online constrained optimal control method, which has been proven to be better than the previously discussed methods. In recent years, it has been widely used in the field of vehicle control, with various applications such as path tracking, collision avoidance, and trajectory planning [24,25,26,27,28]. For instance, Chowdhri et al. proposed an MPC-based approach that considers brake constraints and collision avoidance with the vehicle in front [24], while Chen et al. designed MPC to complete tracking control of 14-DOF vehicles with tire turning angle and road adhesion constraints [25,26]. Igarashi et al. proposed a linearization method to improve vehicle operation efficiency [27], and Wu et al. implemented MPC for path planning of collision avoidance to ensure the stability of driving [28]. However, traditional MPC algorithms require precise system models to improve tracking accuracy, and vehicles are often affected by unknown external disturbances during actual operation, leading to a loss of control system robustness [29]. To address these issues, tube MPC methods have been proposed, such as the robust method by Mata-Machuca et al. [30], which uses a linear feedback control law as an auxiliary control law to improve the robustness of traditional MPC algorithms against unknown factors. However, the offline calculation of the auxiliary control law reduces its convergence speed, making it less robust [31]. In this paper, we propose an SMC-based tube MPC to overcome the limitations of traditional tube MPC. By combining the receding-horizon optimization of MPC with SMC’s strong ability to suppress internal and external disturbances, we significantly improve stability and tracking accuracy compared to other MPC algorithms.

Given the tracking instability problem caused by internal unmodeled uncertainty and external disturbances such as slippery or rough roads during the operation of AV, this paper proposes a robust SMC-based tube MPC method. The main contributions are as follows:

(1) This paper presents a new SMC-based tube MPC strategy for AV trajectory tracking by combining discrete time MPC control strategy and discrete time SMC. The proposed strategy improves control accuracy and robustness.

(2) To enhance the robustness of MPC in the presence of external disturbances and internal modeling uncertainties, we introduce an auxiliary SMC control law to address any bounded disturbances. Experimental results under three different road conditions, namely muddy roads, snowy roads, and icy roads, demonstrate the superior robustness of the proposed method compared to MPC and traditional tube MPC methods.

(3) This paper provides a design method for the constraint inputs of the entire control law, the robust control invariant set of the SMC, and the boundedness of the upper control. The stability of the proposed method is also analyzed.

The remainder of this paper is structured as follows: In Section 3.1, we present the derivation of the vehicle system, while Section 3.2 provides details on the linearization and discretization of the system. The proposed algorithm is introduced in Section 4.1, followed by the design of the nominal MPC controller in Section 4.2, and the stability analysis of the nominal MPC in Section 4.3. In Section 4.4, we present the design of the auxiliary SMC controller, and Section 4.5 analyzes the stability of the auxiliary SMC. Section 4.6 outlines the control flow of the proposed algorithm. Finally, in Section 5, we analyze the results of experiments conducted under different environmental conditions.

## 2. Notations

In this paper, we use R to represent the set of real numbers, $R^{n\times n}$ to represent the set of real n×n matrices, and $R^{n}$ to represent a vector with *n* real number elements. The identity matrix of any dimension is represented by *I*, and diag(·) denotes the diagonal matrix. The Minkowski sum is denoted by the operator ⨁. The Euclidean norm is represented by ${\left|x\right|}^{2}=x^{T}x$, and its weighted norm is denoted by ${\left|x\right|}_{W}^{2}=x^{T}Wx$, where *W* is the weight matrix.

## 3. Problem Formulation

### 3.1. Model Derivation

**Figure 1:**
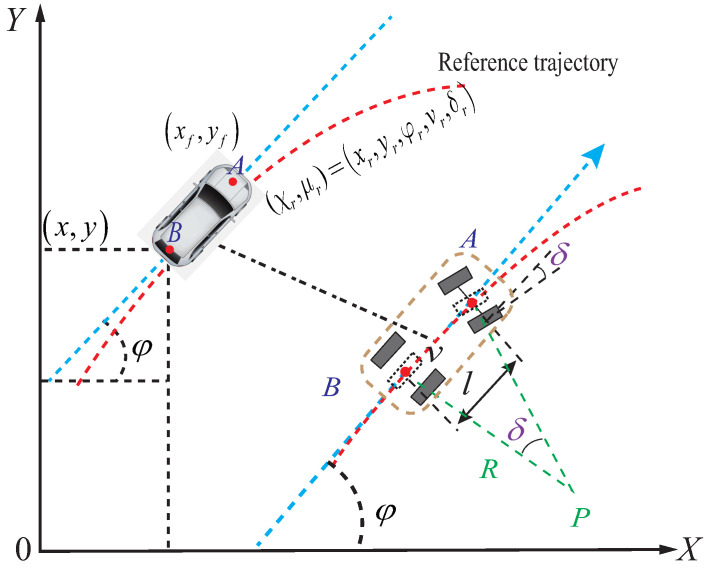
The basic structure of an autonomous vehicle (AV).

This paper considers a front-wheel steering vehicle modeled using the Ackerman steering bicycle model. The kinematic system structure and related symbols of the controlled vehicle are illustrated in Figure 1. In Figure 1, (x,y) is the coordinate of the rear axle axis *B*, $\left(x_{f},y_{f}\right)$ is the coordinate of the front axle axis *A*, *l* is the axle length between the front and rear wheels, *P* is the instantaneous rotation center, and *R* is the rotation radius. The kinematic system of the vehicle can be constructed as follows [32]:

(1) $$\left\{\begin{array}{c} x_{f}=x+l\cos\varphi \\ y_{f}=y+l\sin\varphi \end{array}\right.$$

(2) $$\left\{\begin{array}{c} {\dot{x}}_{f}\sin\left(\varphi +\delta \right)={\dot{y}}_{f}\cos\left(\varphi +\delta \right)\\ \dot{x}\sin\left(\varphi \right)=\dot{y}\cos\left(\varphi \right) \end{array}\right.$$

where δ represents the angle of the front wheel steering, while ${\dot{x}}_{f}$, ${\dot{y}}_{f}$, $\dot{x}$, and $\dot{y}$ represent the speeds of the front and rear wheels in the *X*-axis and *Y*-axis directions, respectively. The yaw angle of the vehicle is denoted by φ, while *v* represents the speed of the vehicle. The yaw rate $\dot{\varphi }$ can be calculated using the relationship between the center coordinates of the rear axle (x,y) and the vehicle speed *v*. The vehicle kinematics can be described by the following equations:

(3) $$\left\{\begin{array}{c} \dot{x}=v\cos\varphi \\ \dot{y}=v\sin\varphi \\ \dot{\varphi }=\frac{v\tan\delta }{l} \end{array}\right.$$

To account for the physical limitations of AV, including maximum speed and steering wheel position, Equation (4) expresses the input constraints. It is essential to ensure stability in the tracking process while applying these constraints.

(4) $$U^{\omega }:=\left\{\begin{array}{cc} v: & v_{\min}\leq v\leq v_{\max}\\ \delta : & \delta_{\min}\leq \delta \leq \delta_{\max} \end{array}\right\}$$

where $U^{\omega }$ denotes the set of control variables constraints. Specifically, $v_{\min}$ and $v_{\max}$ correspond to the minimum and maximum speeds that the vehicle can reach. Meanwhile, $\delta_{\min}$ and $\delta_{\max}$ represent the minimum and maximum steering angles of the vehicle’s front wheels, respectively.

### 3.2. The Linearization and Discretization of the Kinematic AV

Taking into account the impact of actual bounded uncertain disturbance, the kinematic model of the controlled vehicle (3) can be formulated as a general form continuous-time nonlinear system equation, which can be expressed as follows:

(5) $$\dot{\chi }=f\left(\chi ,\mu \right)+\omega$$

The variable $\chi ={\left(x,y,\varphi \right)}^{T}$ represents the state of the vehicle, and $\dot{\chi }={\left(\dot{x},\dot{y},\dot{\varphi }\right)}^{T}$ denotes their corresponding velocities. The input control variable is denoted by $\mu ={\left(v,\delta \right)}^{T}$ and the bounded uncertain disturbance is represented by ω.

The predetermined tracking reference can be represented as $\chi_{r}={\left(x_{r},y_{r},\varphi_{r}\right)}^{T}$, while the desired reference tracking input can be represented as $\mu_{r}={\left(v_{r},\delta_{r}\right)}^{T}$. The reference equation is given by ${\dot{\chi }}_{r}=f\left(\chi_{r},\mu_{r}\right)$. We can simplify the system Equation (5) by expanding it using a Taylor series with respect to the tracking reference, ignoring higher-order terms after the first order.

(6) $$\begin{array}{cc} \dot{\chi }=f\left(\chi_{r},\mu_{r}\right) & +{\left.\frac{\partial f}{\partial \chi }\right|}_{\begin{array}{c} \chi =x_{r}\\ \mu =\mu_{r} \end{array}}\left(\chi -\chi_{r}\right)+{\left.\frac{\partial f}{\partial \mu }\left(\mu -\mu_{r}\right)\right|}_{\begin{array}{c} \chi =\chi_{r}\\ \mu =\mu_{r} \end{array}}+\omega \end{array}$$

Subtracting the reference equation ${\dot{\chi }}_{r}=f\left(\chi_{r},\mu_{r}\right)$ from Equation (6), we can obtain the linearized model as follows:

(7) $$\begin{array}{cc} \dot{\bar{\chi }}= & \left[\begin{array}{c} \dot{x}-{\dot{x}}_{r}\\ \dot{y}-{\dot{y}}_{r}\\ \dot{\varphi }-{\dot{\varphi }}_{r} \end{array}\right]\\ = & \left[\begin{array}{ccc} 0 & 0 & -v_{r}\sin\varphi_{r}\\ 0 & 0 & v_{r}\cos\varphi_{r}\\ 0 & 0 & 0 \end{array}\right]\left[\begin{array}{c} x-x_{r}\\ y-y_{r}\\ \varphi -\varphi_{r} \end{array}\right]+\left[\begin{array}{cc} \sin\varphi_{r} & 0\\ \cos\varphi_{r} & 0\\ \frac{\tan\delta_{r}}{l} & \frac{v_{r}}{l\cos^{2}\delta_{r}} \end{array}\right]\left[\begin{array}{c} v-v_{r}\\ \delta -\delta_{r} \end{array}\right]+\omega \end{array}$$

where $\dot{\bar{\chi }}=\dot{\chi }-{\dot{\chi }}_{r}$, assuming a sampling time of *T*, the above continuous-time system can be discretized as follows:

(8) $$\hat{\chi }\left(k+1\right)=\tilde{A}\hat{\chi }\left(k\right)+\tilde{B}\hat{\mu }\left(k\right)+\omega \left(k\right)$$

$$\begin{array}{cc} & \hat{\chi }\left(k\right)=\chi \left(k\right)-\chi_{r}\left(k\right)=\left[\begin{array}{c} x\left(k\right)-x_{r}\left(k\right)\\ y\left(k\right)-y_{r}\left(k\right)\\ \varphi \left(k\right)-\varphi_{r}\left(k\right) \end{array}\right],\\ & \hat{\mu }\left(k\right)=\mu \left(k\right)-\mu_{r}\left(k\right)=\left[\begin{array}{c} v\left(k\right)-v_{r}\left(k\right)\\ \delta \left(k\right)-\delta_{r}\left(k\right) \end{array}\right], \end{array}$$

$$\tilde{A}=I+T\cdot A=\left[\begin{array}{ccc} 1 & 0 & -Tv_{r}\sin\varphi_{r}\\ 0 & 1 & Tv_{r}\cos\varphi_{r}\\ 0 & 0 & 1 \end{array}\right],$$

$$\tilde{B}=T\cdot B=\left[\begin{array}{cc} T\sin\varphi_{r} & 0\\ T\cos\varphi_{r} & 0\\ T\frac{\tan\delta_{r}}{l} & T\frac{v_{r}}{l\cos^{2}\delta_{r}} \end{array}\right].$$

The input constraint is expressed as Equation (9):

(9) $$\hat{\mu }\left(k\right)\in U^{\omega }$$

The variable ω(k) defined above consists of two parts. The first part represents the internal unmodeled uncertainties of the vehicle kinematics, while the second part corresponds to external disturbances encountered during actual driving. For instance, the vehicle may experience various unknown disturbances on slippery and rough roads due to rainwater or snow accumulation, which can affect the vehicle’s state during motion. Both the unmodeled uncertainties and external disturbances are represented by ω(k)∈W, where $W=\{\omega \left(k\right)\in R^{3},∥\omega (k)∥\leq \omega_{\max}\}$ denotes the set of bounded uncertainties with maximum assumption $\omega_{\max}$.

## 4. The Proposed Robust SMC-Based Tube-MPC Algorithm for AV

### 4.1. The Designing of SMC-Based Tube-MPC Controller

During the sampling interval *T*, the total control input of the actual system is designed as follows:

(10) $$\hat{\mu }\left(k\right)={\hat{u}}^{*}\left(k\right)+\phi \eta \left(k\right)$$

where the optimal control law ${\hat{u}}^{*}\left(k\right)$ is obtained from the optimal cost function (12), subject to the constraint that ${\hat{u}}^{*}\left(k\right)\in U$ and $\hat{\mu }\left(k\right)\in U^{\omega }$, where $U^{\omega }$=U⊕ϕη(k). The adjustable parameter matrix $\phi \in R^{n_{\mu }}$, where $n_{\mu }$ is the dimension of the input. The ancillary tube SMC law η(k) is designed based on an error system described below.

### 4.2. The Designing of the Nominal MPC

Under ideal conditions with no internal or external interference, the nominal system of the AV is given by Equation (11):

(11) $$\hat{\xi }\left(k+1\right)=\tilde{A}\hat{\xi }\left(k\right)+\tilde{B}\hat{u}\left(k\right)$$

where $\hat{\xi }\left(k\right)=\xi \left(k\right)-\chi_{r}\left(k\right)$ is the deviation between the nominal system state and the reference tracking state, and $\hat{u}\left(k\right)=u\left(k\right)-\mu_{r}\left(k\right)$ is the deviation between the input and the reference input.

To achieve a smooth, fast, and accurate tracking of the reference trajectory, a soft constraint approach is adopted to formulate the cost function for optimal control [33]. This technique helps to alleviate the problem of exceeding hard constraint limits caused by frequent acceleration, deceleration, and steering adjustments. Therefore, the cost function is defined as follows:

(12) $$\begin{array}{cc} J(k)= & \sum_{i=1}^{N_{p}}∥\hat{\xi }{\left(k+i|k\right)∥}_{Q}^{2}+\sum_{i=0}^{N_{c}-1}{∥\hat{u}\left(k+i|k\right)∥}_{R}^{2}+\kappa \varepsilon {\left(k\right)}^{2}\\ \; & \;s.t.\;\;\hat{u}\left(k+i|k\right)\in U\\ \; & \;\;\hat{\xi }\left(k+N_{p}|k\right)=0 \end{array}$$

The objective function constraint is $\hat{u}\left(k\right)\in U$, as calculated in Section 4.4, and an additional mandatory constraint $\hat{\xi }\left(k+N_{p}|k\right)=0$ is included in the aforementioned cost function. The constants $N_{p}$ and $N_{c}$ represent the prediction and control horizons, $Q\in R^{n_{\hat{\xi }}\times n_{\hat{\xi }}}$ and $R\in R^{n_{\hat{u}}\times n_{\hat{u}}}$ are weighted diagonal matrices of state variables and input variables, respectively. $n_{\hat{\xi }}$ is the state quantity dimension, $n_{\hat{u}}$ is the input quantity dimension. The relaxation factor ε and the weighting coefficient κ are used to balance the constraints. The goal is to avoid infeasible optimization problems caused by constraints. When the constraints are exceeded, the relaxation variable ε becomes positive and expands the feasible range of $\hat{u}$; otherwise, ε is set to 0. Moreover, to regulate the input energy, a quadratic penalty term is added to the cost function to achieve optimal control.

Then, the optimal control sequence is obtained by solving the constrained optimization problem of the nominal predictive controller at each step.

(13) $$\begin{array}{cc} U^{*}\left(k\right) & =\arg\min\;J(k)\\ \; & ={\left[{\hat{u}}^{*}\left(k\right),{\hat{u}}^{*}\left(k+1\right),\cdots ,{\hat{u}}^{*}\left(k+N_{c}-1\right)\right]}^{T} \end{array}$$

Its corresponding optimal state trajectory and cost function are defined as the symbol ${\left[{\hat{\xi }}^{*}\left(k+1\right),{\hat{\xi }}^{*}\left(k+2\right),\cdots ,{\hat{\xi }}^{*}\left(k+N_{p}\right)\right]}^{T}$ and $J^{*}\left(k\right)$.

After selecting the first item ${\hat{u}}^{*}\left(k\right)$ in Equation (13) as the nominal control signal, it is transmitted to the vehicle body for control. Then, the optimization problem is immediately updated using the newly acquired state, and the process is executed recursively until the control process is completed. This iterative approach ensures that the control system continuously adapts to changes in the system and environment, thereby enhancing the system’s adaptability and robustness.

### 4.3. The Stability Analysis of the Nominal MPC System

In this section, the stability analysis of the nominal system (11) using the proposed MPC algorithm is presented below.

**Theorem 1.**  *Consider the nominal system (11) and the cost function (12) with soft constraints, a positive semi-definite matrix Q, the positive definite matrix R. For ease of calculation and understanding, let $N_{p}=N_{c}=N$. Let the optimal cost function $J^{}\left(k\right)$ at time instant k be the Lyapunov function $V^{}\left(k\right)$. Then, $V^{}\left(k\right)\geq V^{}\left(k+1\right)$ holds at time instant k+1, which implies that the optimal solution ensures the nominal stability of the system (11).*

**Proof.**  The Lyapunov function is defined as follows:

(14) $$\begin{array}{cc} V^{*}\left(k\right) & =\min_{\left[\hat{u}\left(k+i|k\right),\varepsilon \left(k\right)\right]}J\left(k\right)\\ & =\min_{\left[\hat{u}\left(k+i|k\right),\varepsilon \left(k\right)\right]}\sum_{i=1}^{N_{}}∥\hat{\xi }{\left(k+i|k\right)∥}_{Q}^{2}+\sum_{i=0}^{N_{}-1}{∥\hat{u}\left(k+i|k\right)∥}_{R}^{2}+\kappa \varepsilon {\left(k\right)}^{2} \end{array}$$

The input of the nominal system at time instant k+1 can be expressed as

(15) $$\begin{array}{cc} \hat{u}\left(k+i+1|k+1\right)= & [\hat{u}\left(k+1|k+1\right),\hat{u}\left(k+2|k+1\right)\ldots \left.\ldots ,\hat{u}\left(k+N-1|k+1\right),0\right]\\ = & [{\hat{u}}^{*}\left(k+1|k\right),{\hat{u}}^{*}\left(k+2|k\right)\ldots \left.\ldots ,{\hat{u}}^{*}\left(k+N-1|k\right),0\right]\\ \varepsilon (k+1)= & \varepsilon^{*}\left(k\right) \end{array}$$

According to Equation (15), the nominal state of the system at time instant k+1 is expressed as follows:

$$\hat{\xi }\left(k+i+1|k+1\right)={\hat{\xi }}^{*}\left(k+i+1|k\right),i=1,\cdots ,N-1$$

then

(16) $$\begin{array}{cc} \hat{\xi }\left(k+i+1|k+1\right)= & [\hat{\xi }\left(k+2|k+1\right),\hat{\xi }\left(k+3|k+1\right)\ldots \left.\ldots ,\hat{\xi }\left(k+N|k+1\right)\right]\\ = & [{\hat{\xi }}^{*}\left(k+2|k\right),{\hat{\xi }}^{*}\left(k+3|k\right)\ldots \left.\ldots ,{\hat{\xi }}^{*}\left(k+N|k\right)\right] \end{array}$$

It should be noted that in the above relationship (15), the $\hat{u}\left(k+N|k+1\right)=0$ and ${\hat{\xi }}^{*}\left(k+N|k\right)=0$ can be observed by Equation (12). By utilizing Equation (11), we obtain:

(17) $$\begin{array}{cc} \hat{\xi }\left(k+N+1|k+1\right) & =\tilde{A}\hat{\xi }\left(k+N|k+1\right)+\tilde{B}\hat{u}\left(k+N|k+1\right)\\ \; & =0 \end{array}$$

By considering the cost function (14) at time instant *k*, we can derive the relationship between J(k+1) and $V^{*}\left(k\right)$, which is given by Equation (18) below:

(18) $$\begin{array}{cc} J(k+1)= & \sum_{i=1}^{N_{}}∥\hat{\xi }{\left(k+1+i|k+1\right)∥}_{Q}^{2}+\sum_{i=0}^{N_{}-1}{∥\hat{u}\left(k+1+i|k+1\right)∥}_{R}^{2}+\kappa \varepsilon {\left(k+1\right)}^{2}\\ = & \sum_{i=2}^{N_{}}{∥{\hat{\xi }}^{*}\left(k+i|k\right)∥}_{Q}^{2}+\sum_{i=1}^{N_{}-1}{∥{\hat{u}}^{*}\left(k+i|k\right)∥}_{R}^{2}+\kappa \varepsilon^{*}{\left(k\right)}^{2}+{∥\hat{\xi }\left(k+N_{}+1|k+1\right)∥}_{Q}^{2}\\ \; & +{∥\hat{u}\left(k+N_{}|k+1\right)∥}_{R}^{2}\\ = & \sum_{i=1}^{N_{}}{∥{\hat{\xi }}^{*}\left(k+i|k\right)∥}_{Q}^{2}+\sum_{i=0}^{N_{}-1}{∥{\hat{u}}^{*}\left(k+i|k\right)∥}_{R}^{2}+\kappa \varepsilon^{*}{\left(k\right)}^{2}-∥{\hat{\xi }}^{*}{\left(k+1|k\right)∥}_{Q}^{2}-{∥{\hat{u}}^{*}\left(k|k\right)∥}_{R}^{2}\\ = & V^{*}\left(k\right)-∥{\hat{\xi }}^{*}{\left(k+1|k\right)∥}_{Q}^{2}-{∥{\hat{u}}^{*}\left(k|k\right)∥}_{R}^{2} \end{array}$$

It can be seen from the properties of the optimal solution that any solution at the time instant k+1 is not less than the optimal solution $V^{*}\left(k+1\right)$, that is $V^{*}\left(k+1\right)\leq J\left(k+1\right)$.

(19) $$\begin{array}{cc} V^{*}\left(k+1\right) & \leq J(k+1)\\ \; & \leq V^{*}\left(k\right)-{∥{\hat{\xi }}^{*}\left(k+1\right)∥}_{Q}^{2}-{∥{\hat{u}}^{*}\left(k\right)∥}_{R}^{2} \end{array}$$

Finally, note that since ${∥{\hat{\xi }}^{}\left(k+1\right)∥}_{Q}^{2}+{∥{\hat{u}}^{}\left(k\right)∥}_{R}^{2}⩾0$, it follows that $V^{*}\left(k+1\right)⩽V^{*}\left(k\right)$. Therefore, the stability of the nominal system is proven. □

### 4.4. The Designing of the Ancillary SMC

Before designing the auxiliary SMC controller, an error model needs to be established to represent the difference between the nominal system model (11) and the actual system (8) under the influence of internal and external disturbances. The error model can be obtained by substituting Equation (10) into Equation (8), resulting in the following Equation (20):

(20) $$\epsilon \left(k+1\right)=\tilde{A}\epsilon \left(k\right)+\tilde{B}\phi \eta \left(k\right)+\omega \left(k\right)$$

Among them, the error state is represented by $\epsilon \left(k\right)=\hat{\chi }\left(k\right)-\hat{\xi }\left(k\right)=\chi \left(k\right)-\xi \left(k\right)$. The auxiliary tube SMC law ϕη(k) aims to ensure that the trajectory of the error system remains within the invariant set $\Omega_{\mathrm{tube}\;}$ centered on the nominal trajectory. The calculation process of ϕη(k) and $\Omega_{\mathrm{tube}\;}$ is explained in Equations (24) and (25). In other words, SMC laws maintain the AV’s tracking accuracy by keeping the actual system in line with the nominal system.

The discrete-time switching function is established according to the error system function (20) as follows:

(21) $$s\left(k\right)=C_{e}\epsilon \left(k\right)$$

In the above equation, $C_{e}\in R^{1\times n_{\chi }}$ is a constant matrix with $n_{\chi }$ being the dimension of the state variable. Since the disturbance factor ω(k) is unknown and cannot be directly measured, a delay estimation method can be utilized to estimate the disturbances:

(22) $$\begin{array}{cc} \hat{\omega }\left(k\right) & =\epsilon \left(k\right)-\tilde{A}\epsilon \left(k-1\right)-\tilde{B}\phi \eta \left(k-1\right)\\ \; & =\omega (k-1) \end{array}$$

Meanwhile, to further improve the convergence process, the reaching law of the SMC controller is designed as

(23) $$\begin{array}{cc} s(k+1)= & \left(1-qT\right)s\left(k\right)-\frac{\lambda }{\beta +\mathrm{sgn}(s(k))}T{\mathrm{sig}}^{\alpha }\left(s\left(k\right)\right)+C_{e}\sigma \left(k\right) \end{array}$$

where ${\mathrm{sig}}^{\alpha }\left(s\left(k\right)\right)=|s\left(k\right){|}^{\alpha }\mathrm{sgn}\left(s\left(k\right)\right)$, 0<qT<1, 1<λ<β, 0<α<1, σ(k)=ω(k)−ω(k−1). The auxiliary tube SMC law is derived as follows:

(24) $$\begin{array}{cc} \eta (k)= & {\left(C_{e}\tilde{B}\phi \right)}^{-1}\left(\left(1-qT\right)s\left(k\right)-\frac{\lambda }{\beta +\mathrm{sgn}(s(k))}T{\mathrm{sig}}^{\alpha }\left(\left(s\left(k\right)\right)\right)-C_{e}\tilde{A}\epsilon \left(k\right)-C_{e}\hat{\omega }\left(k\right)\right) \end{array}$$

**Lemma 1.**  *If the auxiliary tube SMC law is known as the Equation (24) mentioned above, then the upper limit of the control input can be expressed as*

(25) $$\begin{array}{cc} \Omega_{\mathrm{tube}\;}=\{\eta \left(k\right):∥\eta \left(k\right)∥\leq & ∥{\left(C_{e}\tilde{B}\phi \right)}^{-1}∥\left[∥C_{e}\tilde{A}∥\omega_{\max}+∥C_{e}∥\omega_{\max}+qT∥C_{e}∥\omega_{\max}\right.\\ \; & \left.\left.+\frac{\lambda }{\beta +\mathrm{sgn}(s(k))}T{∥C_{e}∥}^{\alpha }\omega_{\max}{}^{\alpha }+∥C_{e}∥\omega_{\max}\right]\right\} \end{array}$$

**Proof.** 
(26) $$\begin{array}{cc} ∥\eta (k)∥\leq & ∥{\left(C_{e}\tilde{B}\phi \right)}^{-1}∥\left[∥C_{e}\tilde{A}\epsilon \left(k\right)∥\right.+\left|s\left(k\right)\right|+qT|s\left(k\right)|\\ \; & +\frac{\lambda }{\beta +\mathrm{sgn}(s(k))}T\left|{\mathrm{sig}}^{\alpha }\left(s\left(k\right)\right)\right|+∥C_{e}\hat{\omega }\left(k\right)∥] \end{array}$$

Assuming that the switching function and known disturbances are bounded, we can establish the following condition:

(27) $$\begin{array}{cc} ∥\eta (k)∥\leq & ∥{\left(C_{e}\tilde{B}\phi \right)}^{-1}∥\left[∥C_{e}\tilde{A}∥∥\epsilon \left(k\right)∥\right.+\left|s\left(k\right)\right|+qT|s\left(k\right)|\\ \; & +\frac{\lambda }{\beta +\mathrm{sgn}(s(k))}T{\left|\left(s\left(k\right)\right)\right|}^{\alpha }+∥C_{e}∥\omega_{\max}] \end{array}$$

Therefore, the set of auxiliary tube SMC law is bounded by

(28) $$\begin{array}{cc} \Omega_{\mathrm{tube}\;}=\{∥\eta \left(k\right)∥\leq & ∥{\left(C_{e}\tilde{B}\phi \right)}^{-1}∥\left[∥C_{e}\tilde{A}∥∥\epsilon \left(k\right)∥+\left|s\left(k\right)\right|+qT|s\left(k\right)|\right.\\ \; & \left.\left.+\frac{\lambda }{\beta +\mathrm{sgn}(s(k))}T{\left|\left(s\left(k\right)\right)\right|}^{\alpha }+∥C_{e}∥\omega_{\max}\right]\right\} \end{array}$$

Based on the stability analysis in Section 4.5, it can be inferred that |s(k)| is a decreasing function, and $∥\epsilon (k)∥$ is also decreasing. Therefore, we can assume the initial maximum disturbance limit $\omega_{\max}$ to be the upper bound of $∥\epsilon (k)∥$. In other words, $∥\epsilon (k)∥\leq \omega_{\max}$ and $\left|s\left(k\right)\right|\leq ∥C_{e}∥∥\epsilon (k)∥\leq ∥C_{e}∥\omega_{\max}$. This leads to the conclusion in Equation (25). □

**Remark 1.**  *To obtain the nominal constraint of MPC, we use the upper bound of the auxiliary SMC law as a basis and define $U=U^{\omega }⊕\left(-\phi \Omega_{\mathrm{tube}\;}\right)$ [34]. By doing so, the designed QP function (11) of the nominal MPC satisfies the tight constraint U.*

### 4.5. The Stability Analysis of Auxiliary Tube SMC Law

**Theorem 2.**  *For the discrete-time tube error system (20) with an allowable disturbance ω(k), applying the auxiliary tube SMC law (24), we can obtain the following results [35]:*

(1) Sliding mode state s(k) can enter the region Ω within $K^{*}$ steps and remain there indefinitely, where

(29) $$\begin{array}{cc} \Omega & =\{s(k):|s(k)|\leq \rho \\ \; & =\psi \left(\alpha \right)\max\{{\left(\frac{\left(\beta +\mathrm{sgn}\left(s\left(k\right)\right)\right)\sigma^{*}}{\lambda T}\right)}^{\frac{1}{\alpha }},\;\frac{\lambda T}{\left(\beta +\mathrm{sgn}(s(k))(1-qT\right)}\} \end{array}$$

*with*

$$\psi \left(\alpha \right)=1+\alpha^{\frac{\alpha }{1-\alpha }}-\alpha^{\frac{1}{1-\alpha }},$$

$$K^{*}=\left[m^{*}\right]+1\;,$$

$$m^{*}=\frac{s^{2}\left(0\right)-\rho^{2}}{\varrho^{2}},$$

$$\varrho =qT\rho +\left[\psi^{\alpha }\left(\alpha \right)-1\right]\sigma^{*}.$$

*(2) The |s(k)| in the switching function (21) is a decreasing function.*

**Proof.**  The proof process consists of three main steps.

***Step*** **1**.

The reaching law of SMC is given by Equation (23). However, previous studies (references [36,37]) have suggested that non-quadratic Lyapunov functions may offer better performance. In this paper, we use a simple design form of the Lyapunov function, $V\left(k\right)=s^{2}\left(k\right)$, to facilitate calculation and comprehension.

(30) $$\begin{array}{cc} \Delta V(k)= & V(k+1)-V(k)\\ = & -\left(qTs\left(k\right)+\frac{\lambda }{\beta +\mathrm{sgn}(s(k))}T{\mathrm{sig}}^{\alpha }\left(s\left(k\right)\right)-C_{e}\sigma \left(k\right)\right)\\ \; & \times \left(2s\left(k\right)-qTs\left(k\right)-\frac{\lambda }{\beta +\mathrm{sgn}(s(k))}T{\mathrm{sig}}^{\alpha }\left(s\left(k\right)\right)+C_{e}\sigma \left(k\right)\right) \end{array}$$

Next, we consider two cases when s(k)∉Ω.

***Case*** **1**. When s(k)>ρ,

(31) $$\begin{array}{cc} \; & s\left(k\right)>\rho =\psi \left(\alpha \right)\left({\left(\frac{\beta +1}{\lambda T}\right)}^{\frac{1}{\alpha }}\right),\\ \; & qTs\left(k\right)+\frac{\lambda }{\beta +1}Ts^{\alpha }\left(k\right)-C_{e}\sigma \left(k\right)\\ \geq & qTs\left(k\right)+\sigma^{*}\psi^{\alpha }\left(\alpha \right)-\left|C_{e}\sigma \left(k\right)\right|\\ \geq & qT\rho +\left[\psi^{\alpha }\left(\alpha \right)-1\right]\sigma^{*}:=\varrho \end{array}$$

where $1<\psi^{\alpha }\left(\alpha \right)<2$, sgn(s(k))=1, ${\mathrm{sig}}^{\alpha }\left(s\left(k\right)\right)=s^{\alpha }\left(k\right)$, $\sigma^{*}>0$, $\sigma^{*}$ is a constant assumed to exist, making $|C_{e}\sigma \left(k\right)|\leq \sigma^{*}$, so ϱ is also a constant.

On the other hand, it is noted that

(32) $$\begin{array}{cc} \; & s\left(k\right)>\rho =\psi \left(\alpha \right){\left(\frac{\lambda T}{\left(\beta +1)(1-qT\right)}\right)}^{\frac{1}{1-\alpha }} \end{array}$$

Transform Equation (32) as follows:

(33) $$\begin{array}{cc} \; & \left(1-qT\right)s{\left(k\right)}^{1-\alpha }>\psi_{\left(\alpha \right)}^{1-\alpha }\frac{\lambda }{\beta +1}T\\ \; & \left(1-qT\right)s\left(k\right)>\psi_{\left(\alpha \right)}^{1-\alpha }\frac{\lambda }{\beta +1}Ts^{\alpha }\left(k\right)>\frac{\lambda }{\beta +1}Ts^{\alpha }\left(k\right) \end{array}$$

Which means that

(34) $$\begin{array}{cc} \; & s\left(k\right)>qTs\left(k\right)+\frac{\lambda }{\beta +1}Ts^{\alpha }\left(k\right) \end{array}$$

Combining Equations (30), (31) and (34), we can conclude that:

(35) $$\begin{array}{cc} \; & 2s\left(k\right)-qTs\left(k\right)-\frac{\lambda }{\beta +1}Ts^{\alpha }\left(k\right)+C_{e}\sigma \left(k\right)\\ \geq & qTs\left(k\right)+\frac{\lambda }{\beta +1}Ts^{\alpha }\left(k\right)-\left|C_{e}\sigma \left(k\right)\right|\geq \varrho \end{array}$$

In conclusion, we obtain $\Delta V\left(k\right)\leq -\varrho^{2}$.

***Case*** **2**. When s(k)<−ρ,

Based on the above conclusion, we can derive the following relationship (36).

***Step*** **2**.

$$s{\left(k+1\right)}^{2}-s{\left(k\right)}^{2}\leq -\varrho^{2}$$

(36) $$\left\{\begin{array}{c} s{\left(k+1\right)}^{2}\leq s{\left(k\right)}^{2}-\varrho^{2}\\ s{\left(1\right)}^{2}\leq s{\left(0\right)}^{2}-\varrho^{2}\\ s{\left(2\right)}^{2}\leq s{\left(0\right)}^{2}-2\varrho^{2}\\ \vdots \\ s{\left(m\right)}^{2}\leq s^{2}\left(0\right)-m\varrho^{2} \end{array}\right.$$

If $s{\left(m^{*}\right)}^{2}=\rho^{2}$, it indicates that at time $m^{*}$, s(k) reaches the boundary of the region Ω. At the next time step, $K=m^{*}+1$, it enters the switching band.

***Step*** **3**.

Next, it is proved that when s(k) enters the area Ω, it will not exceed the boundary. The definition of Φ is expressed as follows:

(37) $$\begin{array}{c} \Phi =\max\left\{{\left(\frac{\left(\beta +\mathrm{sgn}\left(s\left(k\right)\right)\right)\sigma^{*}}{\lambda T}\right)}^{\frac{1}{\alpha }}\right.,\left.{\left(\frac{\lambda T}{\left(\beta +\mathrm{sgn}(s(k))(1-qT\right)}\right)}^{\frac{1}{1-\alpha }}\right\} \end{array}$$

Let ρ=ψ(α)Φ, considering the following Equations (38) and (39):

(38) $$\left\{\begin{array}{c} {\left(\frac{\left(\beta +\mathrm{sgn}\left(s\left(k\right)\right)\right)\sigma^{*}}{\lambda T}\right)}^{\frac{1}{\alpha }}\leq {\left(\frac{\lambda T}{\left(\beta +\mathrm{sgn}(s(k))(1-qT\right)}\right)}^{\frac{1}{1-\alpha }}=\Phi \\ \sigma^{*}\leq \frac{\lambda }{\left(\beta +\mathrm{sgn}(s(k)\right)}{\left({\left(\frac{\lambda T}{\left(\beta +\mathrm{sgn}(s(k))(1-qT\right)}\right)}^{\frac{1}{1-\alpha }}\right)}^{\alpha }\\ \sigma^{*}\leq \frac{\lambda }{\left(\beta +\mathrm{sgn}(s(k)\right)}T\Phi^{\alpha }=\left(1-qT\right)\Phi \end{array}\right.$$

(39) $$\left\{\begin{array}{c} \Phi ={\left(\frac{\left(\beta +\mathrm{sgn}\left(s\left(k\right)\right)\right)\sigma^{*}}{\lambda T}\right)}^{\frac{1}{\alpha }}\geq {\left(\frac{\lambda T}{\left(\beta +\mathrm{sgn}(s(k))(1-qT\right)}\right)}^{\frac{1}{1-\alpha }}\\ \sigma^{*}=\frac{\lambda T}{\left(\beta +\mathrm{sgn}(s(k)\right)}\Phi^{\alpha }\\ {\left(\sigma^{*}\right)}^{1-\alpha }\geq \frac{\lambda T}{\left(\beta +\mathrm{sgn}\left(s\left(k\right)\right){\left(1-qT\right)}^{\alpha }\right.}\\ \sigma^{*}\leq \left(1-qT\right){\left(\frac{\left(\beta +\mathrm{sgn}\left(s\left(k\right)\right)\sigma^{*}\right.}{\lambda T}\right)}^{\frac{1}{\alpha }}=\left(1-qT\right)\Phi \end{array}\right.$$

The following derivation (40) can be obtained

(40) $$\sigma^{*}\leq \frac{\lambda }{\beta +\mathrm{sgn}(s(k))}T\Phi^{\alpha }\leq \left(1-qT\right)\Phi$$

Since −ρ≤s(k)≤ρ, assuming s(k)=θρ=θψ(α)Φ,−1≤θ≤1, we can obtain:

(41) $$\begin{array}{cc} s(k+1) & =\left(1-qT\right)\theta \rho -\frac{\lambda }{\beta +\mathrm{sgn}(s(k))}T{\mathrm{sig}}^{\alpha }\left(\theta \rho \right)+C_{e}\sigma \left(k\right)\\ \; & \leq \left(1-qT\right)\psi \left(\alpha \right)\theta \Phi -\frac{\lambda }{\beta +\mathrm{sgn}(s(k))}T{\mathrm{sig}}^{\alpha }\left(\psi \left(\alpha \right)\theta \right)\Phi^{\alpha }+\sigma^{*} \end{array}$$

Similarly, we consider two cases when s(k)∈Ω.

***Case*** **1**. When s(k+1)≤ρ.

If ψ(α)θ≥0:

(42) $$s\left(k+1\right)\leq \left(1-qT\right)\psi \left(\alpha \right)\theta \Phi -{\left(\psi \left(\alpha \right)\theta \right)}^{\alpha }\sigma^{*}+\sigma^{*}$$

If ψ(α)θ≥1:

(43) $$s\left(k+1\right)\leq \left(1-qT\right)\psi \left(\alpha \right)\theta \Phi \leq \psi \left(\alpha \right)\Phi =\rho$$

If 0≤ψ(α)θ≤1:

(44) $$\begin{array}{cc} s(k+1) & \leq [1+\psi \left(\alpha \right)\theta -{\left(\psi \left(\alpha \right)\theta \right)}^{\alpha }]\left(1-qT\right)\Phi \\ \; & \leq \left(1-qT\right)\Phi \\ \; & <\psi (\alpha )\Phi =\rho \end{array}$$

If ψ(α)θ≤0, it can be obtained according to the derivation (40):

(45) $$\begin{array}{cc} s(k+1)\leq & -\left(1-qT\right)|\psi \left(\alpha \right)\theta |\Phi +{\left|\psi \left(\alpha \right)\theta \right|}^{\alpha }\left(1-qT\right)\Phi +\sigma^{*} \end{array}$$

If ψ(α)θ≤−1:

(46) $$s\left(k+1\right)\leq \sigma^{*}\leq \left(1-qT\right)\Phi <\psi \left(\alpha \right)\Phi =\rho$$

If −1≤ψ(α)θ≤0:

(47) $$s\left(k+1\right)\leq -\left[|\psi \left(\alpha \right)\theta \right|-{\left|\psi \left(\alpha \right)\theta \right|}^{\alpha }-1]\left(1-qT\right)\Phi$$

By $\left|\psi \left(\alpha \right)\theta \right|-{\left|\psi \left(\alpha \right)\theta \right|}^{\alpha }-1\geq -\psi \left(\alpha \right)$ in Lemma 2, we can obtain $s\left(k+1\right)\leq \left(1-qT\right)\psi \left(\alpha \right)\Phi <\psi \left(\alpha \right)\Phi =\rho$.

***Case*** **2**. When s(k+1)≥−ρ.

According to the definition of Equation (41) and ρ, we can obtain:

(48) $$\begin{array}{cc} s(k+1)\geq & \left(1-qT\right)\psi \left(\alpha \right)\theta \Phi -\frac{\lambda }{\beta +\mathrm{sgn}(s(k))}T{\mathrm{sig}}^{\alpha }\left(\psi \left(\alpha \right)\theta \right)\Phi^{\alpha }-\sigma^{*} \end{array}$$

Based on the derivation in Equation (40), it is clear that

If ψ(α)θ≥1:

(49) $$s\left(k+1\right)\geq -\sigma^{*}\geq -\left(1-qT\right)\Phi >-\rho$$

If 0≤ψ(α)θ<1:

(50) $$\begin{array}{cc} s(k+1) & \geq \left[\psi \left(\alpha \right)\theta -{\left(\psi \left(\alpha \right)\theta \right)}^{\alpha }-1\right]\left(1-qT\right)\Phi \\ \; & \geq -\psi \left(\alpha \right)\left(1-qT\right)\Phi \\ \; & >-\psi (\alpha )\Phi =-\rho \end{array}$$

If −1<ψ(α)θ≤0:

(51) $$\begin{array}{cc} s(k+1) & \geq -\left(1-qT\right)|\psi \left(\alpha \right)\theta |\Phi +{\left|\psi \left(\alpha \right)\theta \right|}^{\alpha }\sigma^{*}-\sigma^{*}\\ \; & \geq -[1+|\psi \left(\alpha \right)\theta |-\left.{\left|\psi \left(\alpha \right)\theta \right|}^{\alpha }\right]\left(1-qT\right)\Phi \\ \; & \geq -\left(1-qT\right)\Phi \\ \; & >-\rho \end{array}$$

If ψ(α)θ≤−1:

(52) $$\begin{array}{cc} s(k+1) & \geq \left(1-qT\right)\psi \left(\alpha \right)\theta \Phi +{\left|\psi \left(\alpha \right)\theta \right|}^{\alpha }\sigma^{*}-\sigma^{*}\\ \; & \geq (1-\left.qT\right)\psi \left(\alpha \right)\theta \Phi \\ \; & >-\psi (\alpha )\Phi =-\rho \end{array}$$

Thus, we can deduce that −ρ≤s(k+1)≤ρ, which indicates that s(k+1)∈Ω. □

**Lemma 2.**  *If $\psi \left(\alpha \right)=1+\alpha^{\frac{\alpha }{1-\alpha }}-\alpha^{\frac{1}{1-\alpha }}$ and 0<α<1, So 1<ψ(α)<2, for any x∈[0,1], we have the following conclusion:*

(53) $$x\psi \left(\alpha \right)-x^{\alpha }\psi {\left(\alpha \right)}^{\alpha }+\psi \left(\alpha \right)-1\geq 0$$

**Proof.**  The function D(x) is defined as

(54) $$D\left(x\right)=x\psi \left(\alpha \right)-x^{\alpha }\psi {\left(\alpha \right)}^{\alpha }+\psi \left(\alpha \right)-1$$

To calculate the minimum value of D(x) in the interval (0,1), we start by noting that D(0)=ψ(α)−1>0 and $D\left(1\right)=\psi \left(\alpha \right)-\psi {\left(\alpha \right)}^{\alpha }+\psi \left(\alpha \right)-1>0$.

Next, we find the critical point of D(x) by setting its derivative to zero:

(55) $$\frac{dD(x)}{dx}=\psi \left(\alpha \right)-\alpha x^{\alpha -1}\psi {\left(\alpha \right)}^{\alpha }=0$$

Solving for *x*, we obtain:

(56) $$x^{*}=\alpha^{\frac{1}{1-\alpha }}\cdot \frac{1}{\psi (\alpha )}$$

Substituting $x^{*}$ into D(x), we get:

(57) $$D\left(x^{*}\right)=\alpha^{\frac{1}{1-\alpha }}-\alpha^{\frac{\alpha }{1-\alpha }}+\psi \left(\alpha \right)-1=0$$

Finally, we compare the values of D(0), D(1), and $D(x^{*})$ and conclude that the minimum value of D(x) in the interval (0,1) is zero. This completes the proof. □

### 4.6. The SMC-Based Tube MPC Algorithm

**Figure 2:**
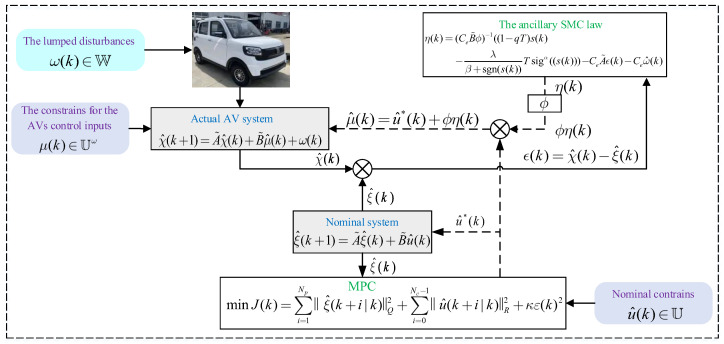
The proposed robust SMC-based tube MPC algorithm.

The proposed robust SMC-based tube MPC algorithm is depicted in Figure 2. To aid comprehension of the controller’s design and application, we present the workflow of the proposed SMC-based tube MPC method in Figure 2, with detailed explanations of the working sequence provided in **Step 1–4**:

**Step 1**: The actual system state $\hat{\chi }\left(k\right)$, which is subject to bounded disturbances ω(k), is decomposed into the nominal system state $\hat{\xi }\left(k\right)$ and error system state ϵ(k).

**Step 2**: The nominal system state $\hat{\xi }\left(k\right)$ is predicted by linearizing the reference trajectory, and the MPC optimization algorithm is employed to compute the nominal control ${\hat{u}}^{*}\left(k\right)$.

**Step 3**: The error system state ϵ(k) is controlled by SMC using a sliding mode state and reaching law to obtain the auxiliary control law ϕη(k). The auxiliary control law is designed to overcome the impact of uncertain disturbances, such as rough and slippery roads, during operation and achieve approximate convergence of the actual system state $\hat{\chi }\left(k\right)$ to the nominal system state $\hat{\xi }\left(k\right)$.

**Step 4**: The nominal MPC control input ${\hat{u}}^{*}\left(k\right)$ and the auxiliary tube SMC law ϕη(k) are combined to form the final actual control input $\hat{\mu }\left(k\right)$, which is used to track the predetermined path of the vehicle in the presence of uncertain disturbances.

The aforementioned process is executed in a receding horizon until path tracking is completed. Overall, this four-step approach offers a comprehensive solution to the challenges of robust lateral control in the presence of disturbances and shows promising results for real-world AV applications.

## 5. Experiments and Analysis

**Figure 3:**
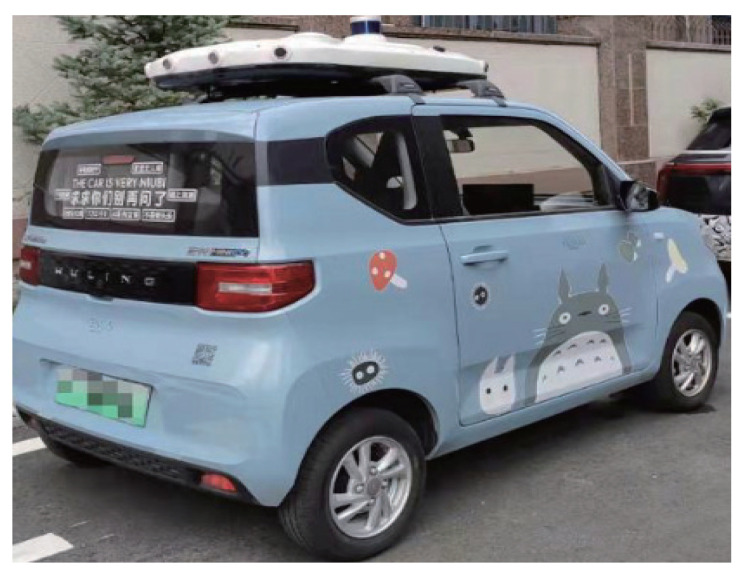
The experimental AV.

To verify the efficacy of the proposed robust SMC-based tube MPC algorithm, an experimental study will be conducted using the Wuling-MiniEV vehicle, which has been retrofitted with CAN-BUS control technology, as depicted in Figure 3. The system leverages full CAN-BUS control and adheres to automotive-grade standards, with wire control techniques enabling features such as front-wheel drive and electro-hydraulic driving brakes. The auto-drive system provides access to the bottom executive control interface and can be integrated with sensors such as lidar, radar, high-precision integrated navigation and positioning systems, and cameras, enabling multi-scene automatic driving applications. To evaluate the stability of the proposed SMC-based tube MPC algorithm, we compare its performance with other classic methods such as LQR [16], traditional MPC [33] and traditional tube MPC [30]. The experimental test road spans a total length of 400 m, consisting of straight road segments, curves, and right-angle turns. The vehicle tracking reference speed is set at 16 km/h, with the tracking instability effect being tested on muddy, snowy, and icy roads.

### 5.1. Muddy Road

Driving on a muddy road section presents a significant challenge for self-driving cars, resulting in delayed control behavior and path tracking overshooting. Based on multiple test situations, we set the maximum disturbance of the controller to (58):

(58) $$\omega_{\max}=10$$

To facilitate comparison, Table 1 presents the parameters of the proposed SMC-based pipeline MPC algorithm on muddy roads, while Table 2 provides the setting parameters of the MPC and LQR controllers.

**Figure 4:**
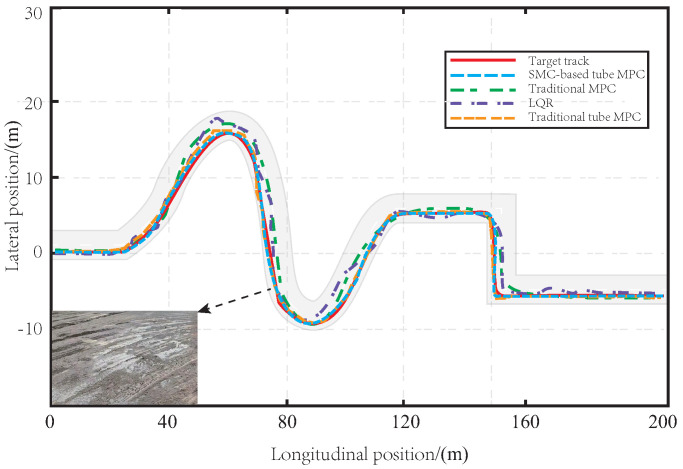
The path tracking effect on the muddy road.

In Figure 4, a comparison of the tracking effect is presented for the proposed method, traditional tube MPC, LQR, and MPC on muddy road sections. The red curve represents the reference trajectory that is set. The tracking accuracy of each method is shown by a dotted line, with the proposed method in blue, the MPC method in green, the traditional tube MPC method in orange, and the LQR method in purple. Since the LQR solves the control law offline, the vehicle body has significant limitations when facing the interference of muddy road sections in the tracking process, resulting in the worst tracking effect. The MPC method can control the vehicle to track the predetermined path through real-time solutions under the influence of muddy road surfaces, but the tracking accuracy is still poor, and its robustness to disturbance is not good. Although the traditional tube MPC algorithm has a good tracking effect, the offline linear feedback control law is selected for the auxiliary control law, which is inferior to the method proposed in this paper in terms of convergence speed and robustness. As SMC is good at resisting bounded interference, the method proposed in this paper can more effectively resist the interference caused by muddy roads than traditional methods, and the tracking effect is more accurate and smooth. This is mainly because our robust SMC-based tube MPC method combines the advantages of MPC and SMC, and has strong robustness in the face of uncertain interference in the actual tracking process, resulting in more accurate tracking.

**Figure 5:**
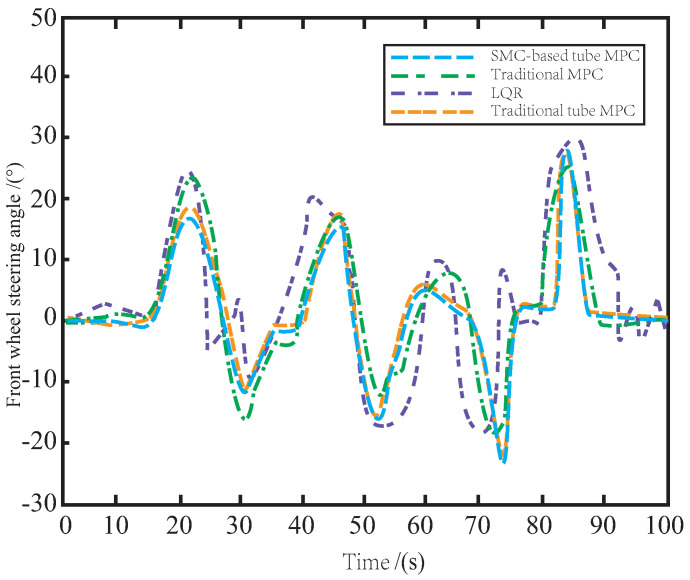
The variation of front wheel angle on the muddy road.

Figure 5 illustrates the steering angles of the front wheels under different control algorithms on muddy roads. It can be observed that LQR-generated control laws cannot stabilize the vehicle body, leading to steering instability. The MPC method exhibits relative stability but still suffers from overshoot caused by muddy roads and internal uncertainties. In contrast, the proposed method has the smoothest tracking process, consumes the least input energy, and achieves the most accurate reference tracking. This is attributed to the SMC-based tube MPC algorithm that enhances the AV’s robustness to external interference and improves its stability compared to the traditional tube MPC. By utilizing the auxiliary SMC feedback control law, the proposed method has the least redundant actions and guarantees the most accurate and smooth tracking of the reference trajectory.

**Figure 6:**
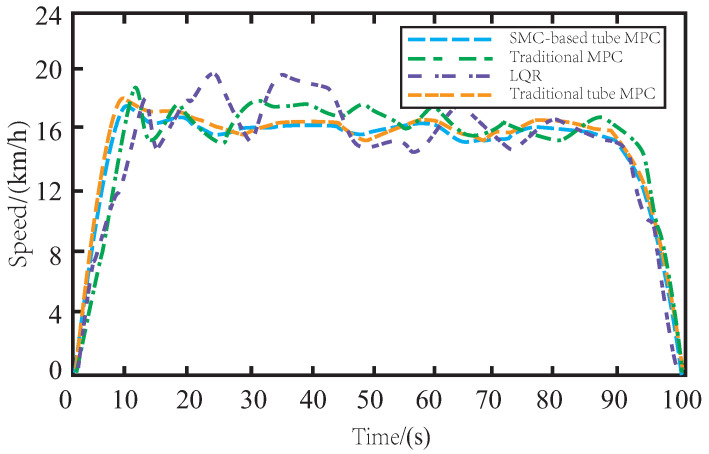
The variation of speed on the muddy road.

Figure 6 shows the speed changes of AV under different control algorithms on muddy roads. The speed of LQR-controlled AV varies significantly during tracking, mainly due to the lack of robustness of the offline control law. Similarly, the MPC method also exhibits frequent speed changes due to the drag caused by muddy roads. By incorporating the SMC algorithm, the proposed SMC-based tube MPC method ensures the smooth changes in AV speed during driving and smooth rise and fall of vehicle speed during cornering. Moreover, it has a simple and fast response time and exhibits robustness to external noise disturbance and parameter ingestion. Compared to the traditional tube MPC, the proposed method ensures smoother speed changes during driving and improves the accuracy and stability of AV control.

### 5.2. Snowy Road

The tracking process is significantly affected by snow-covered roads due to the increased likelihood of sideslip and resistance, particularly during turns, leading to larger deviations from the reference path. The controller is designed to mitigate these effects, with disturbance parameters specified in Equation (59).

(59) $$\omega_{\max}=15$$

**Figure 7:**
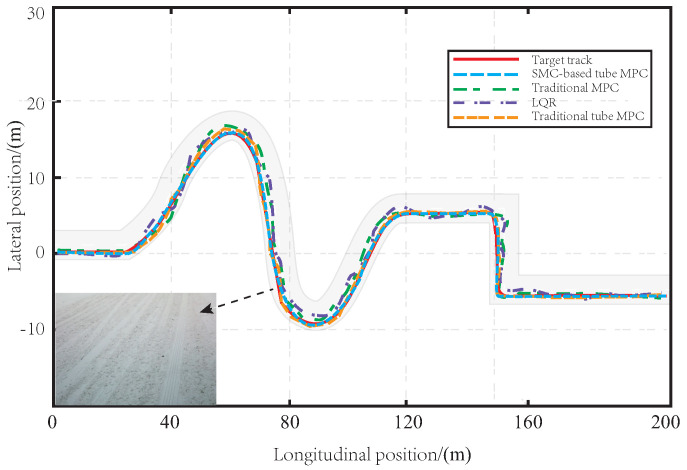
The path tracking effect on the snowy road.

The parameters used for the proposed method on snowy roads are presented in Table 3, while the parameter settings for the MPC and LQR controllers are shown in Table 4:

Figure 7 shows that snow has a significant impact on the tracking performance of LQR and MPC methods. The lack of robustness in these methods leads to large deviation errors during turns, with some even exceeding the lane centerline, posing potential safety risks to the vehicle. While the traditional tube MPC method is effective, it still suffers from errors when dealing with snowy roads. In contrast, the proposed SMC-based tube MPC method uses an auxiliary SMC law that improves robustness and has excellent anti-interference capabilities. Even under the influence of tire sideslip caused by snowy roads, the proposed method can accurately track the reference path with a stable turning process.

**Figure 8:**
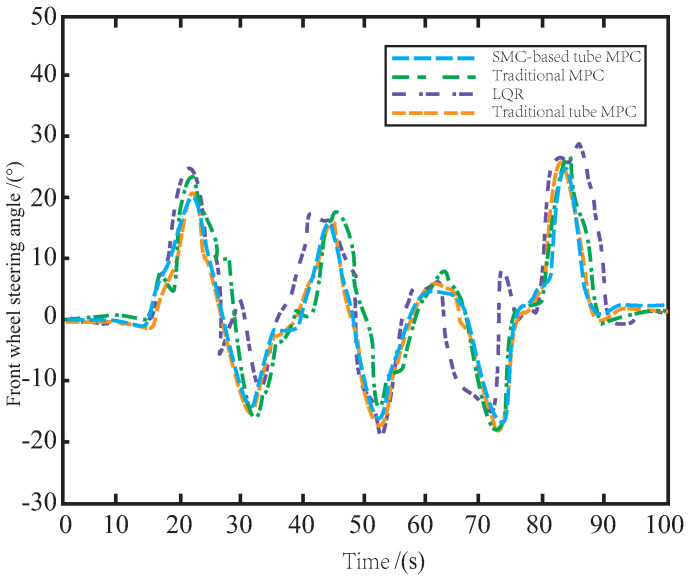
The variation of front wheel angle on the snowy road.

As depicted in Figure 8, the control performance of LQR and MPC methods on the snowy road is still suboptimal due to the influence of snow, resulting in complex and redundant changes in the front wheel angle, which may cause potential safety risks such as skidding during driving. To address this issue, the proposed SMC-based tube MPC method modifies the traditional tube MPC by incorporating an auxiliary SMC control law, which enhances the stability of the front wheel steering. With the excellent anti-interference ability of SMC, the impact of snow on the road surface is rapidly suppressed and fed back to the system, reducing the impact of snow on the side slip of the vehicle body. As a result, the AV can still accurately track the reference path while ensuring safe driving.

**Figure 9:**
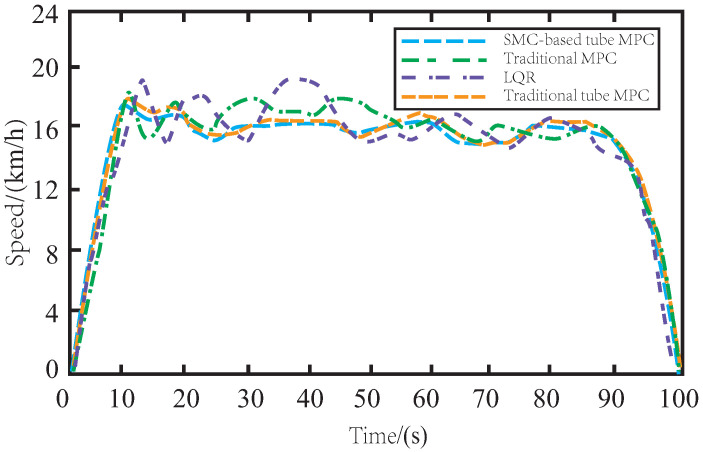
The variation of speed on the snowy road.

In Figure 9, when driving on a snow-covered road, the AV is subject to more unknown disturbances during the tracking process due to side slip and resistance caused by snow. While LQR and MPC methods struggle to overcome the tire side slip, resulting in frequent speed changes, the proposed SMC-based tube MPC method remains stable and can maintain a steady change of speed, outperforming the traditional tube MPC method.

### 5.3. Icy Road

When driving on icy roads, vehicles are more prone to sideslipping and the road surface is typically rougher. The Equation (60) displays the controller’s disturbance parameter configuration.

(60) $$\omega_{\max}=20$$

Table 5 displays the parameters of the proposed method utilized on icy roads, while Table 6 exhibits the parameter settings for the traditional MPC and LQR controllers.

**Figure 10:**
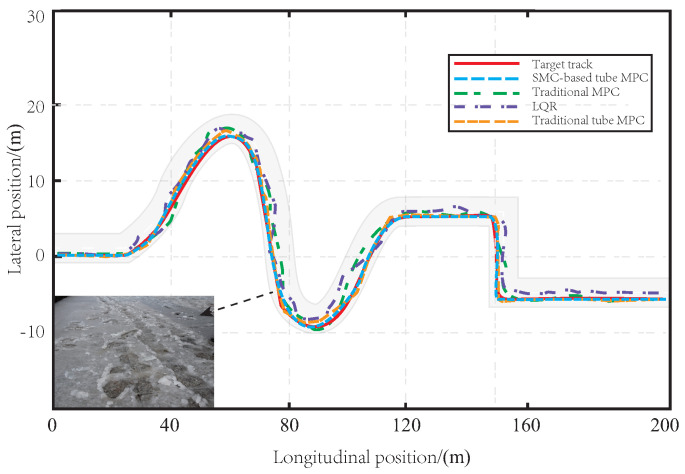
The path tracking effect on the icy road.

As depicted in Figure 10, the LQR and MPC controllers exhibit poor tracking performance on icy road surfaces due to side slips and bumps, particularly when maneuvering turns. The traditional tube MPC demonstrates better performance in handling bumps and side slips, but the controller is subject to higher pressure on icy roads, making it more prone to reaching its limit. By incorporating SMC, the proposed tube MPC approach effectively mitigates the impact of icy conditions on the controller, leading to a more stable and accurate tracking performance with greater robustness.

**Figure 11:**
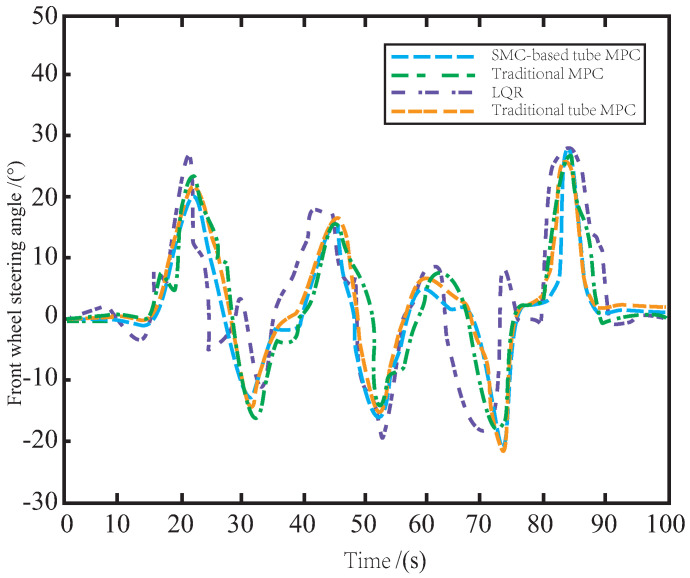
The variation of front wheel angle on the icy road.

As illustrated in Figure 11, when controlled by LQR and MPC, the AV’s front wheels exhibit excessive motion due to side slip and jitter caused by bumps and icy road surfaces, leading to vibrations and unstable driving behavior. By leveraging the auxiliary SMC method, the proposed tube MPC approach outperforms the traditional tube MPC in terms of disturbance suppression and update speed in the disturbance feedback loop. This results in reduced occurrence of front wheel jitter and enables safe and smooth operation of the AV on icy roads.

**Figure 12:**
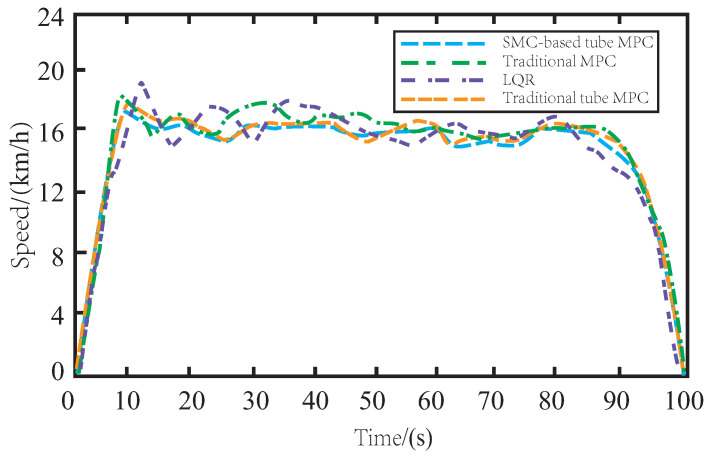
The variation of speed on the icy road.

In Figure 12, it is evident that skidding on icy roads poses a more significant challenge for the AV in terms of maintaining consistent driving speed during tracking. The presence of side slips and bumps further complicates the task of speed control. The insufficient robustness of LQR and MPC controllers makes it difficult to achieve stable changes in vehicle speed. While the traditional tube MPC approach yields satisfactory results due to the inclusion of auxiliary control methods, the proposed SMC-based tube MPC method with improved auxiliary control feedback can further enhance driving comfort by ensuring even smoother changes in AV speed on icy roads.

**Figure 13:**
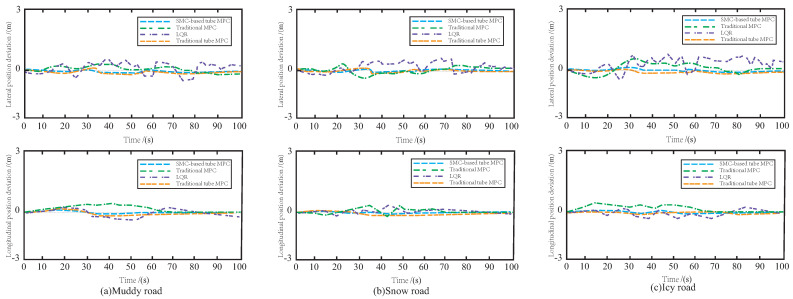
The variation of tracking error on the three roads.

Figure 13a–c illustrate the lateral and longitudinal errors of the four control methods on three different road surfaces, namely, muddy, snowy, and icy roads. The LQR and MPC methods exhibit large deviations in both the horizontal and vertical directions, indicating poor robustness. However, the addition of an auxiliary control law to overcome external disturbances can significantly improve the controller’s performance. The traditional tube MPC method displays high tracking accuracy with small horizontal and vertical errors. Nevertheless, the proposed SMC-based tube MPC effectively suppresses sideslip and drag effects in various road environments, further enhancing control accuracy and stability. This method employs a more robust SMC as the auxiliary control law, featuring a simple structure and fast computation speed, enabling the AV to smoothly and safely track the reference path while ensuring accuracy.

After testing on three types of roads, the average cycle time of the proposed algorithm is shown in Table 7. It can be seen that due to the low dimension of the system, the calculation efficiency of nominal MPC is very fast and remains at about 38 ms, while the calculation efficiency of SMC is even higher. The minimum time is only about 2 ms, and the overall algorithm cycle calculation time takes about 42 ms, which meets the real-time requirements of AV in path tracking. After the path tracking test on three roads, the tracking errors of the four methods are shown in Table 8. Due to the different disturbance effects brought by the three different roads, the LQR tracking error is the largest, about 10%. The effect of traditional MPC is average, with an average error of 5%. After adding an auxiliary control law, the robustness of the controller can be significantly improved. The average error of the traditional tube MPC is about 3.5%, and the SMC-based tube MPC proposed in this paper has further improved the auxiliary control law, resulting in the best performance with an average error of only 2.5%.

In summary, the proposed SMC-based tube MPC algorithm offers smooth steering control, high precision, good stability, and strong adaptability to external disturbances and internal unmodeled uncertainties. It meets the real-time requirements of the actual operation process and provides the best tracking performance during path tracking in challenging conditions such as mud, vibration, ice, and snow. The effectiveness of the proposed control method is demonstrated by its ability to handle any emergencies that occurred during the experiment. Therefore, the algorithm proves to be a promising solution for lateral control in AV.

## 6. Conclusions

This paper presents a novel approach for lateral control of autonomous vehicles by proposing a robust SMC-based tube MPC method. The main objective of this method is to address the tracking overshoot and instabilities that arise from internal unmodeled uncertainties and external disturbances. The design of the proposed method combines discrete-time computational MPC and tube SMC laws, which provide stability and robustness. The nominal MPC is employed to achieve control accuracy with a predictive control law, while the discrete tube SMC law ensures vehicle stability in the presence of disturbances. The proposed method has demonstrated remarkable results, maintaining a tracking error of around 2.5%, which is significantly lower than previous methods. Further research is required to investigate the performance of the proposed method under different road conditions and vehicle speeds, such as strong crosswinds, rain, and high-speed driving. Overall, this SMC-based robust tube MPC method has the potential to improve the lateral control performance of autonomous vehicles in challenging driving scenarios. 

## Figures and Tables

**Table 1:** SMC-based tube MPC.

Nominal MPC	Auxiliary SMC
$N_{P}=N_{C}=8$	$\phi ={\left(0.14,0.23\right)}^{T}$
κ=6	$C_{e}=\left(0.7,0.4,0.3\right)$
Q=diag{1,1,0.5}	q=5
R=diag{0.1,0.1}	λ=3
$v_{\max}=20;\delta_{\max}=30$	β=7

**Table 2:** MPC and LQR.

MPC	LQR
$N_{P}=N_{c}=12$	Q=diag{2,2,2.5}
κ=9	R=diag{1,1,3}
Q=diag{1.5,1.5,1}	
R=diag{0.2,0.15}	
$v_{\max}=20;\delta_{\max}=30$	

**Table 3:** SMC-based tube MPC.

Nominal MPC	Auxiliary SMC
$N_{P}=N_{C}=9$	$\phi ={\left(0.14,0.23\right)}^{T}$
κ=7	$C_{e}=\left(0.7,0.45,0.32\right)$
Q=diag{1,1,0.5}	q=5
R=diag{0.1,0.1}	λ=3
$v_{\max}=0.4;\delta_{\max}=18$	β=7

**Table 4:** MPC and LQR.

MPC	LQR
$N_{P}=N_{c}=14$	Q=diag{3,3,2.5}
κ=11	R=diag{2,2,3}
Q=diag{1.5,1.5,1}	
R=diag{0.2,0.15}	
$v_{\max}=0.4;\delta_{\max}=25$	

**Table 5:** SMC-based tube MPC.

Nominal MPC	Auxiliary SMC
$N_{P}=N_{C}=8$	$\phi ={\left(0.14,0.23\right)}^{T}$
κ=6	$C_{e}=\left(0.8,0.5,0.3\right)$
Q=diag{1,1,0.5}	q=5
R=diag{0.1,0.1}	λ=3
$v_{\max}=20;\delta_{\max}=30$	β=7

**Table 6:** MPC and LQR.

MPC	LQR
$N_{P}=N_{c}=12$	Q=diag{2,2,3}
κ=10	R=diag{1.5,1.5,2}
Q=diag{1.5,1.5,1}	
R=diag{0.2,0.15}	
$v_{\max}=20;\delta_{\max}=30$	

**Table 7:** Average computing time.

Method	Average Computing Time
SMC-based tube MPC	42 (ms)
Nominal MPC	4 (ms)
Auxiliary SMC	38 (ms)

**Table 8:** Average tracking error.

Method	Average Tracking Error
SMC-based tube MPC	2.5%
Traditional tube MPC	3.5%
MPC	5%
LQR	10%

## Data Availability

The data used to support the findings of this study are available from the corresponding author upon request.
